# Logic Circuits Featuring Organic Electrochemical Transistors: What is the Logic Behind OECTs in Logic?

**DOI:** 10.1002/advs.202514448

**Published:** 2025-10-30

**Authors:** Lorenzo Travaglini, Kristina Fidanovski, Damia Mawad

**Affiliations:** ^1^ School of Materials Science and Engineering UNSW Sydney Sydney New South Wales 2052 Australia; ^2^ Soft Transducers Laboratory Ecole Polytechnique Fédérale de Lausanne (EPFL) Rue de la Maladière 71b Neuchâtel CH‐2000 Switzerland

**Keywords:** bioelectronic, circuit, complementary, logic, OECT

## Abstract

For bioelectronic applications, the optimal electronic device is one capable of effectively interfacing with living systems by coupling electronic conduction and the ionic conduction which underlies signal transmission in physiological environments. In this context, the organic electrochemical transistor (OECT), which has a conjugated polymer‐based active channel, is a promising device since the conjugated polymer can feature mixed ionic‐electronic conduction in aqueous electrolytes. Having gained a solid understanding of the OECT and its operational fundamentals, research has now shifted towards integrating the OECT as a building block in logic circuits; which are featured in this review due to the advanced functionalities they enable. First, a brief overview of OECT's key parameters and advantages are presented. Next, the integration of OECTs in unipolar and complementary configurations is discussed. This is followed by examples demonstrating the implementation of these circuits along with their defining characteristics. Finally, a brief overview highlights the key challenges that must be addressed to enable the development of more complex OECT‐based circuitry capable not only of transducing bio‐signals but also processing them. OECT‐based circuits represent a solution for applications requiring soft and flexible electronic devices and provide a valid alternative to inorganic technologies at the bio‐interface.

## Graphical abstract

For bioelectronic applications, organic electrochemical transistors (OECTs), with their mixed ionic and electronic conduction, will be invaluable for interfacing with living systems. This review examines the latest research, which has shifted towards integrating OECTs in logic circuits capable of transducing and processing bio‐signals. As such, OECT‐based circuits are ideal candidates for bioapplication solutions and represent an alternative to inorganic technologies.

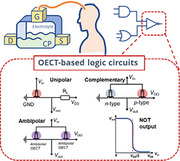

## 1 Introduction

The interface of electronics with biological systems has marked a pivotal milestone in medical treatment, with a range of diseases now routinely treated with implantable electronic devices, hereafter referred to as bioelectronics. Examples of such devices include cardiac pacemakers,^[^ ^1^ ^]^ implantable prosthetic devices,^[^ ^2^ ^]^ cochlear implants,^[^ ^3^ ^]^ deep brain and spinal cord stimulators,^[^ ^4^ ^]^ and retina implants.^[^ ^5^ ^]^ What made these devices a reality was the commercialization of the transistor in the 1950′s, followed by advances in solid‐state devices and microelectronics. The s‐ubsequent size reduction of the transistor and its incorporation into integrated circuits (ICs) enabled complex functionalities such as locally and partially processing the electrophysiological signal before it is transmitted to external processing units for more complex analysis.

Commercial bioelectronic devices are typically based on metal oxide semiconductor field effect transistors (MOSFETs),^[^ ^6^ ^]^ which are built on rigid silicon substrates that enable the manufacturing of devices with reduced dimensions and advanced functionalities.^[^ ^7^ ^]^ However, silicon substrates pose a series of challenges when operating in the physiological environment, despite their irrefutable benefits.^[^ ^8^ ^]^ First, their rigid nature does not comply with the soft nature of biological tissues, leading to a foreign body response and encapsulation of the device in some applications. Second, media permeation in the bulk of the device, where the electrical components are enclosed, causes undesirable parasitic currents. Encapsulation of the device is often employed to prevent surface oxidation and moisture permeation; however, this markedly reduces the interface with living tissues.^[^ ^9^, ^10^ ^]^ Third, integrated circuits and mechanical parts typically adhere to a conventional engineering paradigm, characterized by rigid, planar, and heavy electronic blocks. These attributes hinder the seamless integration of such devices with human tissues, often leading to concerns about invasive surgical procedures. Traditionally, addressing this issue primarily involved reducing the size of the device components, though this came with trade‐offs such as increased fabrication complexity and diminished mechanical stability, especially on flexible or nonplanar substrates.

The discovery in 1977 of the semiconducting properties of polyacetylene, a conjugated polymer (CP),^[^ ^11^ ^]^ followed by the development of a plethora of other CPs, ushered in a new era of semiconductor materials that are not only organic but also more biocompatible and flexible. This has led to the emerging field of organic bioelectronic devices with promising benefits over traditional inorganic electronics. The soft nature of organic materials provides better mechanical compatibility with biological systems, enhancing their integration at the biointerface.^[^ ^12^, ^13^, ^14^ ^]^ Organic materials can be readily printed or otherwise processed and can thereby be easily embedded into flexible substrates^[^ ^15^, ^16^ ^]^ that can conform to the tissue surface. Further, their characteristic feature of mixed ionic‐electronic conduction enables seamless communication with biological systems, closely mirroring the natural signalling processes within the body. The organic field effect transistor (OFET)^[^ ^15^, ^16^, ^17^ ^]^ and the organic electrochemical transistor (OECT) are prime examples of how CPs can be used as the active component in bioelectronic devices.^[^ ^18^, ^19^, ^20^, ^21^ ^]^ OECTs are particularly distinguished by their unique operating mechanism: unlike conventional transistors that rely solely on capacitive effects, OECTs operate through ion–electron coupling in wet conditions, making them exceptionally well‐suited for physiological applications. In light of this distinctive operational characteristic, this review provides a comprehensive overview of recent advances in circuits incorporating exclusively OECTs as their building blocks and presents a thorough examination of their applications and design considerations in logic circuits. We first briefly introduce the OECT device architecture, its operational mechanism, and key parameters. We then compare OECTs to OFETs and discuss why OECTs offer distinct advantages in bioelectronic applications. This is followed by presenting typical logic circuit design approaches, including unipolar and complementary configurations. We review examples and strategies implemented to realize OECT‐based logic circuits. We then consider examples where advanced or atypical OECT architectures are leveraged to attain circuits with unique or enhanced properties. Finally, we conclude with a final commentary on the future of OECT‐based logic circuits.

## 2 The Organic Electrochemical Transistor (OECT)

**Figure 1:**
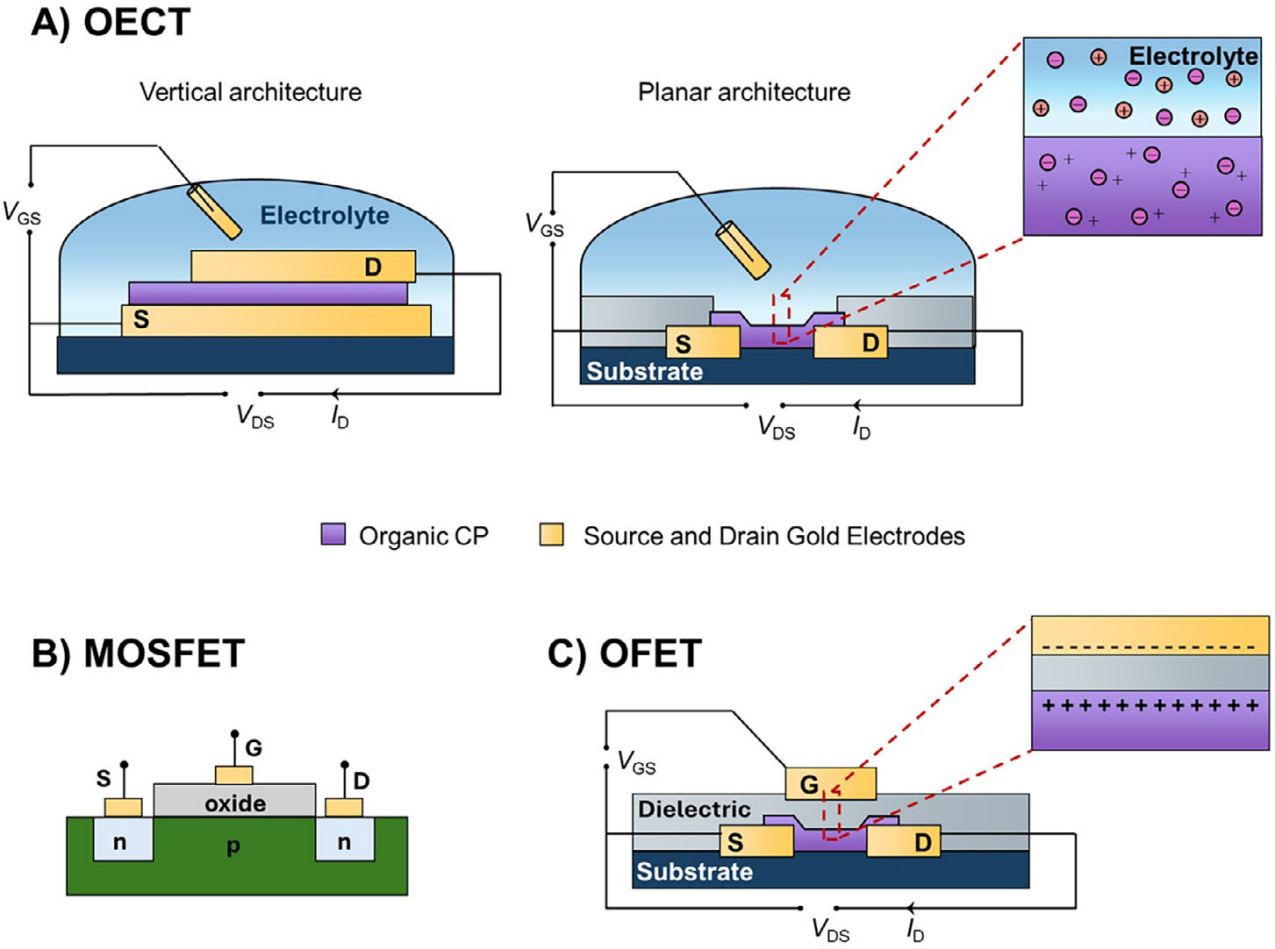
Examples of device architectures and gating mechanisms. A) Vertical (left) and planar (right) architectures of OECTs operating in an electrolyte, where the gate modulates the channel by ion injection/extraction (volumetric doping). B) MOSFET architecture based on a conventional silicon field‐effect transistor with an oxide gate dielectric and purely electronic gating. C) OFET with a solid dielectric, where charge accumulates at the semiconductor/dielectric interface. Insets highlight the ionic (OECT) versus electrostatic (MOSFET/OFET) control of channel conductance. S: source; D: drain; G: gate.

First developed by White et al.,^[^ ^22^ ^]^ the OECT has three terminals: a source, a drain, and a gate. Typical materials for these electrodes include metal electrodes for source and drain, and a non‐polarizable electrode such as Ag/AgCl for the gate, although polarizable electrodes like platinum or gold have also been employed as co‐planar gates^[^ ^23^ ^]^ or in sensing applications that require functional gate modification.^[^ ^24^ ^]^ The CP, which bridges the source and drain terminals, serves as the active channel of the OECT and is in contact with a solid or liquid electrolyte (**Figure** **1**A).

The gate is also in contact with the electrolyte but physically separated from the channel. By varying the relative position of the source, drain, and gate, different configurations of OECTs can be achieved, influencing the device performance.^[^ ^25^ ^]^ Among the various configurations, the most common OECT architecture is having the source and drain in the same plane (planar OECT), bridged by the CP channel material, with two possible gate arrangements: i) the gate immersed in the electrolyte above the channel (top‐gated OECT, Figure 1A) or (ii) the gate patterned in the same plane as the source and drain (coplanar‐gated OECT). Alternatively, the source and drain can be stacked vertically with the CP sandwiched in between, forming what is known as a vertical OECT (vOECT, Figure 1A). The gate can be either top‐gated or coplanar‐gated. The vOECT is emerging as a critical architecture for developing logic circuits, because its vertical stacking enables the fabrication of channels with very short lengths, resulting in enhanced amplification and fast switching capabilities as well as compact footprints suitable for fabricating high‐density arrays.

In all OECT configurations, the input voltage at the gate electrode (*V*_G_) can be used to tune the current between the source and drain, which represents the OECT's output. The active channel conducts a drain current (*I*_D_) when a potential difference between the source and drain electrodes (*V*_DS_) is applied. When a voltage is applied at the gate electrode, the current modulation results from the ingress of the ions from the electrolyte into the active channel, which alters its redox state and changes its doping level.^[^ ^26^ ^]^ Depending on the initial redox state of the channel material, the operational mechanism of an OECT can be either described as depletion mode or accumulation mode. In depletion mode, the channel material initially is in its conductive state and becomes electrochemically de‐doped upon the application of *V*_G_, resulting in reduced conductivity. In contrast, accumulation mode describes an OECT that has a channel material in its semiconducting state that switches to a conductive (electrochemically doped) state when *V*_G_ is applied. These electrochemical processes are initiated when the applied voltage reaches the threshold voltage (*V*_Th_), a key parameter defined as the minimum *V*_G_ required to activate the respective operational mode. Other key parameters that describe the performance of an OECT device include transconductance (*g*_m_), charge carrier mobility (*µ*), and volumetric capacitance (C*) (**Table** **1**). *g*_m_, which is calculated as $\frac{\partial I_{D}}{\partial V_{G}}$, defines the amplification capability of the device and its sensitivity. *µ* and C* are properties of the channel material, with *µ* being a transport property that describes how fast electrons or holes move in the channel material, and C* describes how efficiently the channel material can store charge per unit volume through ion exchange and doping/de‐doping processes. Performance of the OECT can also be characterized by its *I*_ON_/*I*_OFF_ ratio, *I*_ON_ being the maximum drain current when the OECT is in its ON state and *I*_OFF_ being the minimum drain current in the OFF state. A high *I*_ON_/*I*_OFF_ ratio indicates efficient electrochemical doping/dedoping and better signal sensitivity. The response time (*τ*) is also a critical performance parameter, describing how fast the channel current changes with the applied gate voltage. It is comprised of the switching ON time (*τ*_ON_), which is the time taken for the channel to reach a conductive state, and the switching OFF time (*τ*_OFF_), which corresponds to the time for the channel material to return to its less conductive state. Finally, stability, under intermittent and continuous switching, should be evaluated to gain insights into the suitability of the OECT device for applications that require long‐term operational times.

**Figure 2:**
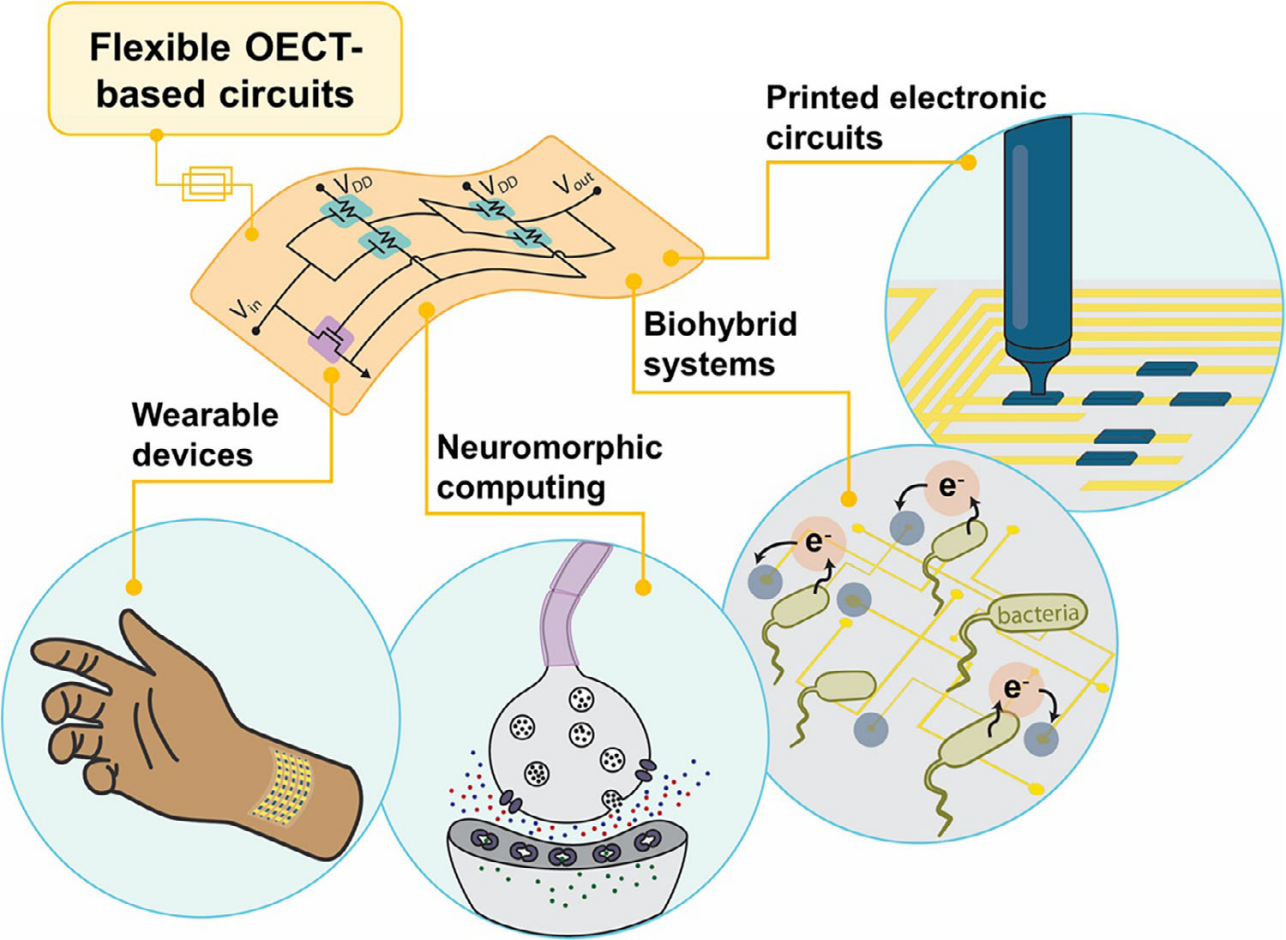
Application landscape for flexible OECT‐based circuits. Schematic overview of key directions: printed electronic circuits enabled by additive manufacturing; wearable devices with soft, skin‐conformal front ends; neuromorphic computing that performs local, low‐voltage signal processing; and biohybrid systems where living components interface with OECT circuitry to sense and compute.

*OECT* versus *OFET*: Similar to a MOSFET or an OFET (Figure 1B,C), the OECT acts as a switch where the current flowing in the active channel (output) can be interrupted or restored with an input voltage at the gate electrode. However, the OECT differs from the OFET in that the gate electrode is separated from the CP channel by the electrolyte instead of a thin layer of a dielectric material. Notably, this architecture offers numerous advantages over that of the OFET when applied to biological systems. First, the high capacitance that results from having the electrolyte between the gate and the active channel allows the OECT to behave as an optimal amplifier with high transconductance, where big changes in drain current in the active layer can be triggered by small voltage inputs at the gate electrode. Second, the operation of OECT in contact with an electrolyte makes it inherently compatible with biological tissues where ion transport dominates physiological activities. A distinctive feature of the OECT is its high sensitivity to ions, enabling the detection and transduction of ionic currents such as electrophysiological signals. This property is particularly valuable in bioelectrochemistry, ion sensing, and other applications where precise monitoring of ion‐based signals is essential. Additionally, the low operating voltage of OECTs (< 1 V) compared to OFETs offers a significant advantage in that it minimizes the risk of electrochemical side reactions that could damage the device and induce cytotoxicity. It also results in low power consumption, critical for bioelectronic implants designed for long‐term applications where battery life is critical. These characteristics make the OECT suitable for applications at the interface with biology,^[^ ^27^, ^28^, ^29^ ^]^ and, as such, serve as a fundamental building block in bioelectronic circuits, often functioning as a transducer or sensor due to its intrinsic signal amplification and high signal‐to‐noise ratio.^[^ ^30^, ^31^, ^32^ ^]^ Combined with low‐voltage operation, solution processability, and compatibility with flexible or unconventional substrates, these features have enabled the development of OECT‐based device architectures such as printed electronic circuits,^[^ ^33^ ^]^ active and hybrid bioelectronic devices,^[^ ^34^, ^35^, ^36^ ^]^ neuromorphic components, and organic memories (**Figure** **2**).^[^ ^37^, ^38^ ^]^

Expanding the potential of flexible organic bioelectronics, further advancements can be achieved by using the OECT in ICs that not only detect electronic signals but also process them. While early research focused on single‐device performance and basic circuit elements, current efforts are increasingly directed toward building more complex logic circuits, composed of multiple OECTs to unlock advanced functionalities. As the logic gate is the fundamental building block of modern electronic components, this expansion of capabilities broadens the scope of applications in organic bioelectronics, allowing for enhanced signal manipulation. This progress is further accelerated by the ability to fabricate entire OECT circuits using additive manufacturing techniques, which streamlines production and enables rapid prototyping on flexible substrates. Additive manufacturing techniques have demonstrated the feasibility of fabricating low‐cost, scalable OECTs on flexible substrates with direct patterning of active channels, conductors, insulators, and electrolytes. A recent review on additive manufacturing maps the methods and integration strategies for OECTs, including logic circuits, sensor arrays, and active matrices, while discussing materials/process trade‐offs and scalability challenges.^[^ ^39^, ^40^ ^]^ For example, scalable photolithography has been leveraged to fabricate a high‐sensitivity (2620 mV dec^−1^) OECT‐based ion sensing circuit with a polarizable gate electrode.^[^ ^41^ ^]^ An alternative additive method is the high‐precision micro‐dispensing technique, shown to yield high‐performance monolithic fully printed OECT‐based amplifiers capable of amplifying the electrooculogram signal.^[^ ^42^ ^]^ Recent examples of screen‐ and inkjet‐printed electrochemical logics underscore the role of additive manufacturing in scaling OECT logic and sensing toward large‐area, low‐cost systems.

## 3 Taxonomy of Logic Circuit Configurations

OECTs can be integrated with other circuit elements to enable the construction of logic circuits with signal modulation, amplification, or gating functionalities (**Table** **2**). The current research aims to incrementally increase circuit complexity by improving device design and integration strategies. Such integration supports the development of monolithic circuits based on OECTs, targeting specific use cases across the fields of bioelectronics and low‐power electronics. ICs are essential for implementing complex logic operations, allowing multiple gates and circuit components to coexist on a single substrate. Logic circuits can be classified as unipolar or complementary logic circuits, with the inverter, which acts as a NOT gate, representing the most basic logic circuit. An inverter operates with rail‐to‐rail switching capability, enabling full voltage swing between the supplied voltages. Its working principle is based on biasing the inverter at its transition region, where the voltage transfer curve exhibits the steepest slope. This point is known as the trip point and corresponds to the specific input voltage (*V*_in_) at which output switching occurs. The steep transition region is critical for signal amplification, as small changes in the input signal produce large output voltage (*V*_out_) swings, resulting in high voltage gain. The voltage gain, defined as the ratio of change in output voltage to change in input voltage ($\frac{\partial V_{out}}{\partial V_{\mathrm{in}}})$, quantifies this amplification capability. Coupled with the sharp switching characteristics, this amplification mechanism enables efficient signal processing with minimal noise and low power consumption. Here, we highlight how the OECT is incorporated as a building block into the various architectures of inverters.

### 3.1 The Unipolar Inverter Configuration

**Figure 3:**
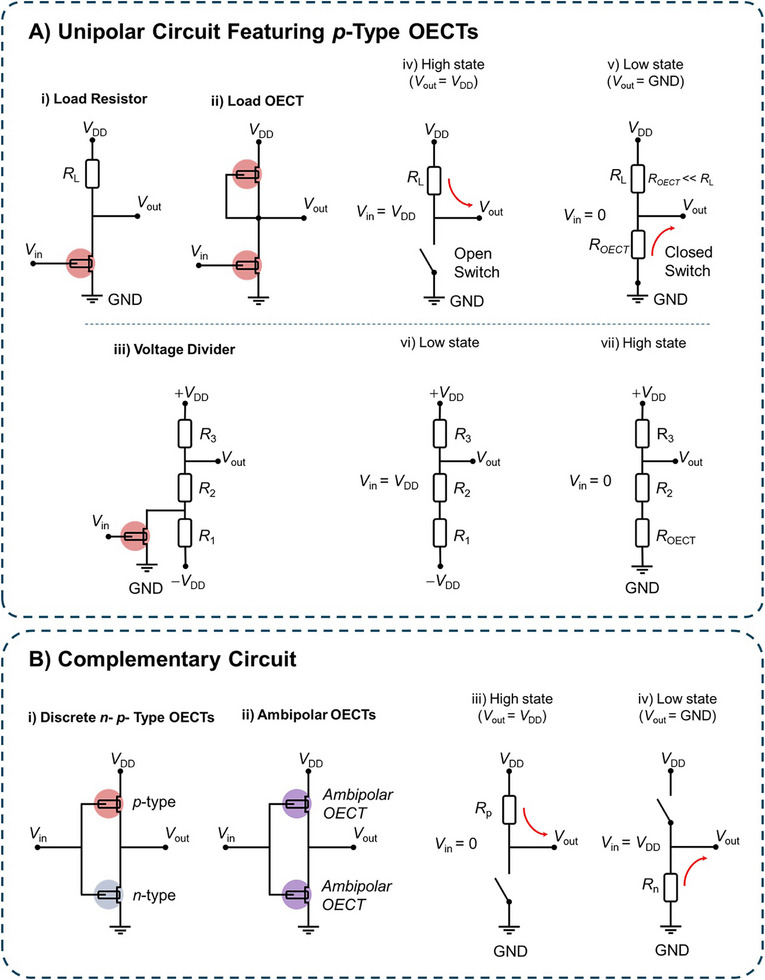
Examples of circuit configurations. A) Unipolar inverter circuit configurations featuring a *p*‐type depletion mode OECT connected in series with a load resistor (*R*_L_) (i), a load OECT (*R*_OECT_) (ii), and a three‐resistor voltage divider (iii), with the relative circuit schematics showing the circuits operating at *V*_in_ = *V*_DD_ (iv, vi) and *V*_in_ = 0 V (v, vii). C) Complementary inverter circuit configurations featuring *n‐* and *p‐*type OECTs (i) and two ambipolar OECTs (ii) connected in series, with relative circuit schematics showing the circuit operation for *V*_in_ = 0 V (iii) and *V*_in_ = *V*_DD_ (iv), respectively.

Of the two types of logic circuit configurations, the unipolar is the simpler to execute. A unipolar logic circuit features only one type of transistor; that is, the material of the OECT active channel is exclusively either an *n*‐type (majority electron carriers) or *p*‐type (majority hole carriers) CP. The output voltage is typically modulated by connecting resistors to achieve the desired logic functionality. For instance, in the unipolar inverter shown in **Figure** **3**A(i), an OECT is connected to a passive load resistor (*R*_L_), the drive voltage (*V*_DD_) is applied at the load component, and *V*_out_ is measured between the two components.

The transistor with *V*_in_ at its gate acts as a switch. For example, in the case of a *p*‐type transistor operating in depletion mode, when *V*_in_ is high, i.e., close to *V*_DD_, the OECT is in the OFF state, pulling *V*_out_ to *V*_DD_ (Figure 3A(iv)). On the other hand, when *V*_in_ is low, i.e., close to zero, the OECT is in the ON state and *V*_out_ is pulled close to ground (GND) since *R*_L_ >> *R*_OECT_ (Figure 3A(v)). The optimized response for a load resistor is achieved when the load resistance is double the OECT resistance in its ON state. Architectures that involve an OECT connected to three resistors, typically arranged in series between a positive and a negative *V*_DD_ (Figure 3A(iii)), form OECT‐based circuits configured as voltage dividers. This design enables greater flexibility in tuning *V*_out_ by the choice of the load resistors and the drive voltage. As shown in Figure 3A(iii), the OECT is connected in parallel with the first resistor (*R*_1_) near −*V*_DD,_ and the circuit can exist in two states, which is characteristic of an inverter. The first state corresponds to the low *V*_out_ state (Figure 3A(vi)), which occurs when the transistor is turned OFF, leading to a significant increase in the OECT resistance *R*_OECT_. As a result, *R*_OECT_ becomes much larger than *R*_1_, effectively behaving like an open circuit and thus can be neglected. In this case, *V*_out_ is +*V*_DD_ × (*R*_2_ + *R*_1_) / (*R*_3_ + *R*_2_ + *R_1_*) when −*V*_DD_ is ground.^[^ ^43^, ^44^ ^]^ The second state corresponds to the high *V*_out_ state and occurs when the transistor is turned ON (Figure 3A(vii)), resulting in a small resistance in the parallel resistor such that it can be neglected, and only the resistance of the transistor (*R*_OECT_) is considered. In this case, *V*_out_ is +*V*_DD_ × (*R*_2_ + *R*_OECT_) / (*R*_3_ + *R*_2_ + *R*_OECT_) when −*V*_DD_ is ground. Logic circuits that involve a voltage divider are often used to enable straightforward tuning and optimization of the output voltage by simply changing the value of the resistors connected to the OECT or altering the drive voltage ±*V*_DD_. Another design, which is similar to the load resistor architecture, is one that has a load OECT instead of the resistor (Figure 3A(ii)). The gate of the load OECT is either connected to its source or drain, depending on the type of OECT and its mode of operation. In this configuration, the load OECT has a dynamic nonlinear resistance, and this nonlinear response shapes *V*_out_. This case has the benefit of reduced power consumption relative to the load resistor architecture due to the dynamic nature of the load OECT, which can be ON or OFF, whereas the load resistor is consistently ON and dissipating power.

While the simplicity of the unipolar design is appealing, a downside of using only one type of OECT in this configuration is that once the OECT is triggered to its ON state, current flows through the circuit, leading to a significant increase in power consumption. This elevated power draw can result in undesirable heating of the components, potentially affecting their performance, reliability, and longevity.

### 3.2 The Complementary Inverter Configuration

An alternative to the unipolar design is the complementary configuration, which typically leverages the unique characteristics of both *n*‐ and *p*‐type OECTs connected in series in between *V*_DD_ and GND (Figure 3B(i)). *V*_in_ is applied at the gate of each OECT, and the *V*_out_ is measured in between the OECTs. The *n*‐ and *p*‐type transistors ideally should have matching transport properties to ensure symmetric and reliable switching behaviour, which is essential for proper logic functionality. When one OECT is ON, the other is OFF, effectively minimizing power consumption during logic operations and offering a solution for low‐power applications. This is because power dissipation occurs only briefly during the transient switching of the OECTs between ON and OFF states. The input voltage is selected to ensure that one transistor is conducting while the other is non‐conducting, achieving clear switching behaviour. When *V*_in_ is low, close to zero, the *p*‐type transistor is ON, and the *n*‐type transistor is OFF, pulling *V*_out_ close to *V*_DD_ (Figure 3B(iii)). On the other hand, when *V*_in_ is high, close to *V*_DD_, the *p*‐type transistor is in the OFF state and the *n*‐type transistor is ON, pulling *V*_out_ close to GND (Figure 3B(iv)). As a result, the voltage output swings between the drive voltage *V*_DD_ (*p‐*OECT ON/*n‐*OECT OFF) and GND (*p‐*OECT OFF/*n‐*OECT ON).

As noted earlier, a critical requirement for obtaining a reliable inverter circuit and consequently more complex logic circuits is that the *n*‐ and *p*‐type transistors must have matching electronic performances such as mobility, threshold voltage, and capacitance. This poses a challenge for building a complementary circuit from OECTs, as until recent years, *n*‐type materials lagged behind, typically exhibiting lower carrier mobility and environmental stability than their *p*‐type counterparts, making it difficult to match their electronic characteristics in inverter designs.^[^ ^45^, ^46^ ^]^ Furthermore, while some *n*‐type CPs, such as conjugated ladder‐type polymers, show good stability^[^ ^47^ ^]^ in aqueous environments, achieving a comparable performance across a broader range of *n*‐type polymers remains a major limitation for their application in bioelectronics.^[^ ^48^, ^49^, ^50^ ^]^ To avoid water splitting, these polymers must operate within a narrow electrochemical window below ≈1.2 V. This requires *n*‐type polymers to have sufficiently low LUMO energy levels to prevent unwanted reduction reactions with water or dissolved oxygen. However, designing materials that combine low LUMO levels with good stability, processability, and electronic performance remains difficult, despite some significant advances made recently.^[^ ^51^ ^]^ As a result, *n*‐type polymers with few exceptions often face greater limitations compared to *p*‐type counterparts in bioelectronic device applications. Fortunately, another possible design of complementary‐like architectures features a pair of ambipolar OECTs (Figure 3B(ii)), in which electrons and holes are transported with comparable mobilities within the applied gate voltage range. Achieving ambipolar performance typically requires active materials with narrow bandgaps (< 2 eV), enabling efficient transport of both carrier types. Such materials are often synthesized by copolymerizing electron‐rich and electron‐deficient heteroaromatic units. This synthetic design results in materials with two distinct conductive regions, one for holes and one for electrons, separated by an OFF state (ON–OFF–ON). This dual‐mode conductivity allows precise modulation of the transistor's behaviour, making ambipolar OECTs suitable for versatile and compact logic circuit designs. More recently, CPs that exhibit three reversible switching states (OFF–ON–OFF) within an electrochemical window compatible with aqueous electrolytes (< 1.2 V) such as polyaniline (PANI) and poly(benzimidazobenzophenanthroline) (BBL), are being exploited as single‐material OECTs in complementary circuits. Understanding and characterizing the inverter circuit thoroughly serves as a crucial foundation for the manufacturing and design of more complex logic gates, such as NAND, NOR, and XOR. These gates, with their unique combinations of inputs and outputs, enable the construction of increasingly intricate circuitry, paving the way for the implementation of sophisticated logical operations and digital systems, as will be discussed in the following sections.

## 4 Unipolar Circuits: Implementation

Unipolar circuits have been successfully implemented in a variety of complex configurations. These circuits offer simpler fabrication and design steps compared to complementary systems due to their use of only one OECT type, making them attractive for specific applications despite some limitations in power efficiency. In this section, we focus on the fabrication aspects of unipolar circuits, examining strategies to optimize device performance and integration. Understanding these approaches is essential to advancing scalable and reliable organic bioelectronic systems based on unipolar OECT architectures.

### 4.1 Unipolar Circuits: Load Resistor Type

**Figure 4:**
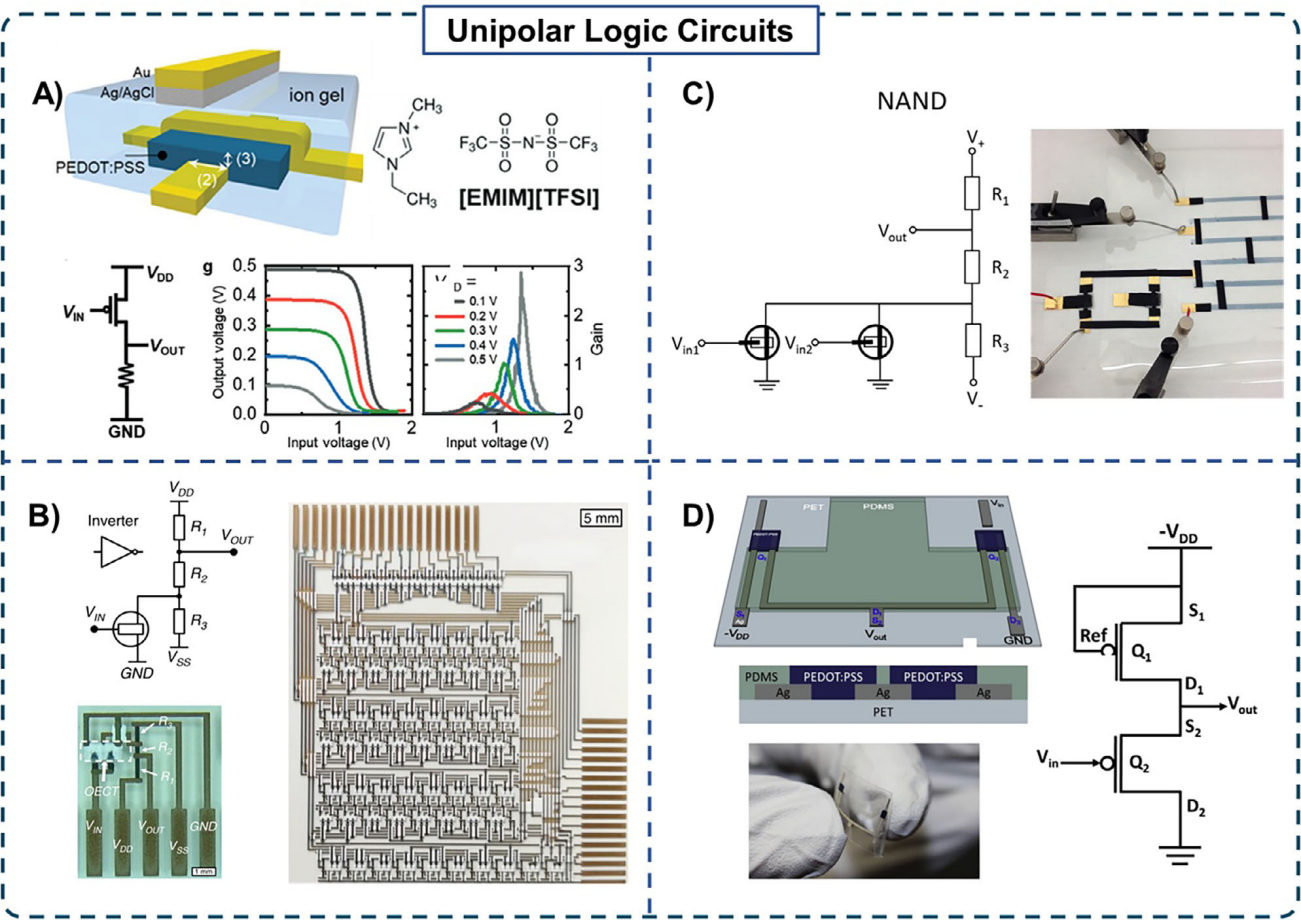
A) Schematic of a vertical OECT used in series with a load resistor, with voltage transfer curves and inverter gain output demonstrating its reliable operation as a NOT logic gate. Reproduced with permission.^[^ ^58^ ^]^ Copyright 2023, Wiley. B) Schematic and optical image of a PEDOT:PSS‐based inverter as well as a seven‐bit shift register composed of 114 OECTs, which was printed using the same screen‐printing methodology. Reproduced with permission.^[^ ^59^ ^]^ Copyright 2019, Springer Nature. C) Schematic and image of a NAND circuit built from a flexible, self‐standing, adhesive composite based on PEDOT:PSS and nanofibrillated cellulose. Reproduced with permission.^[^ ^60^ ^]^ Copyright 2016, IOP. D) 3D rendering, cross‐section, and circuit diagram of a 3D printed unipolar inverter featuring two PEDOT:PSS OECTs on a flexible PET substrate with an image of the same circuit being flexed. Reproduced with permission.^[^ ^61^ ^]^ Copyright 2019, Elsevier.

Recent unipolar designs that combine one OECT connected in series with a load resistor are typically used as voltage amplifiers because the OECT acts as a variable resistor that modulates *V*_out_ based on *V*_in_.^[^ ^43^, ^52^, ^53^, ^54^ ^]^ One example is the unipolar OECT‐based circuit with a channel material made of the *p*‐type polymer p(g2T‐TT), a glycolated polythiophene derivative known for its high mixed ionic–electronic conductivity and enhanced stability in aqueous environments. An electrophysiological sensor composed of a p(g2T‐TT) OECT connected in series with a single 30 kΩ load resistor, with a capacitor added to remove the DC component and maximize AC sensitivity, was successfully able to take low‐amplitude electroencephalography (EEG) measurements.^[^ ^54^ ^]^ The circuit had a voltage gain of 30, which allowed for its clinical application in reliably recording EEG signals in the range 10–100 µV, demonstrating progress towards more sensitive and clinically relevant OECT circuits. Another example is a PEDOT:PSS‐based OECT amplifier configured with a resistive load of 10 kΩ.^[^ ^52^ ^]^ In this circuit, the voltage was measured across the resistor as a function of input frequency. This setup allowed the direct conversion of current modulation into a voltage signal, where changes in gate potential translated into voltage fluctuations across the load resistor. Using a sinusoidal gate input of 10 mV, the circuit demonstrated a maximum voltage gain of ≈12 up to 1 kHz, which the authors emphasized could allow for the amplification of low‐power biological signals at the measurement site, reducing the effect of noise during signal transfer. Subsequently, biosignal acquisition was demonstrated using a similar system to record electrocardiographic signals in a human subject with high sensitivity, underscoring its utility in medical diagnostics.^[^ ^53^ ^]^ We highlighted these studies, despite being primarily examples of amplifiers, to demonstrate the versatility of unipolar OECT circuits with a load resistor. With their simple architecture, they show how design adjustments, such as operating regime and circuit configuration, can tailor the performance of the circuit to specific applications. Additionally, studies based on ion‐gated transistors connected in series with a load resistor demonstrate the potential of this circuit architecture for basic logic functions.^[^ ^55^, ^56^, ^57^, ^58^ ^]^ The NOT logic function of a PEDOT:PSS vOECT with an ionic electrolyte and top‐gated with a solid‐state Ag/AgCl electrode was successfully demonstrated by connecting the vOECT in series with a load resistor (**Figure** **4**A).

The logic inverter operated reliably and as expected, with the gain varying as a function of *V*_DD_, achieving a maximum gain of ≈3 at *V*_DD_ = 0.5 V.^[^ ^58^ ^]^ Similarly, a printed ion‐gel‐gated P3HT transistor was connected in series with a 20 kΩ printed PEDOT:PSS resistor to fabricate an all‐organic printed circuit. Voltage characteristics measurements revealed sharp switching between −1.5 V (“0” state) and 0 V (“1” state) as the input voltage was swept between −1 V and 0 V. The device exhibited a peak gain of 7 with negligible hysteresis, demonstrating consistent and reliable low‐voltage switching performance.^[^ ^56^ ^]^ These studies provide the foundation for further exploration of unipolar OECT circuits with a load resistor in the development of functional logic inverters.

### 4.2 Unipolar Circuits: Voltage Divider Type

Circuits made of a single OECT in conjunction with a voltage divider comprising three resistors connected in series offer the advantage of allowing precise control over the output voltage by selecting appropriate resistor values and the applied drive voltage. One of the first reported examples of a unipolar inverter of this type was fabricated from a PEDOT:PSS based OECT connected to three PEDOT:PSS resistors, resulting in a circuit largely comprised of the same material.^[^ ^62^ ^]^ By incorporating an additional OECT in either parallel or series configuration with the existing transistor, the NAND and NOR logic functions were successfully demonstrated. Furthermore, by connecting five of these inverters in cascade, a ring oscillator was created, showcasing a classical circuit commonly employed for generating clock signals in digital circuits, but which can also be used to characterize the device's switching speed. In this case, the device took ≈10 s to switch between ON and OFF. This research was one of the opening forays into the versatility and potential utility of OECTs in the development of complex logic circuits and integrated systems.

Towards the goal of complex, scalable, high‐resolution logic circuits, various deposition technologies have been explored, starting with simple desktop thermal inkjet printing.^[^ ^63^ ^]^ Using the thermal print head to deposit and pattern both PEDOT:PSS and a water‐based electrolyte on a flexible substrate, an all‐inkjet printed device was achieved. As an extension of the unipolar voltage divider inverter, both NOR and NAND gates were also demonstrated. This method offers circuit designers a straightforward path from concept to prototype, and small‐scale production of a series of flexible electrochemical devices. The authors highlighted that more advanced printing systems could further enhance the flexibility and advantages of inkjet printing, emphasizing the need for improved inkjet‐printable electrolytes.

High‐resolution nanoimprint lithography has been used to great effect to significantly enhance the ON/OFF current ratio (≈1 × 10^4^) of PEDOT:PSS OECTs by reducing the size of the transistor. With feature sizes as small as 10 µm, this approach enabled precise control over the transistor dimensions and substantial improvement in the differentiation between the ON and OFF states.^[^ ^64^ ^]^ This advancement enabled the successful development of a functional voltage divider (6, 16.6, and 40 MΩ) type inverter that exhibited a 3.8 gain, as well as a NAND gate, opening the door towards miniaturized OECTs as key building blocks in integrated circuits.

Building on earlier printing approaches, screen printing has also been demonstrated as an alternative printing method to fabricate all screen‐printed OECT‐based logic circuits on flexible substrates. This has been demonstrated on a PET substrate where the inverter circuit consisted of an OECT and a voltage divider having 3 resistors (25.4, 76.6, and 101.1 kΩ) all printed from PEDOT:PSS, obtaining a maximum gain of 3.3.^[^ ^65^ ^]^ Despite the modest gain, the devices were highly uniform and stable, enabling reliable logic circuits like NAND gates, flip‐flops, and 2‐bit shift registers to be fabricated with consistent output across over 380 OECTs. Using similar screen printing methods, unipolar NAND logic circuits based on vOECTs connected in series with three screen‐printed resistors (45, 25, and 30 kΩ) have also been demonstrated.^[^ ^66^ ^]^ Operating at low voltages (≈1 V), the circuits showed high yield and were a compact design. The same technology enabled more complex circuits, including a 2‐to‐4 decoder (14 OECTs) and a 4‐to‐1 multiplexer (18 OECTs), highlighting the scalability of printed OECT logic for low‐cost electronics. Additionally, these screen‐printing principles have been extended even further, culminating in the successful combination of 114 OECTs in one circuit (Figure 4B).^[^ ^59^ ^]^ Firstly, the basic logic circuits such as NOT and NAND gates were developed using PEDOT:PSS OECTs. The optimal values for the resistors (40.5, 14, and 30.5 kΩ) were pre‐determined by simulations based on a 5 V drive voltage. Using a combination of 29 of the resulting NAND and NOT logic circuits, a seven‐bit shift register was developed, composed of 114 OECTs in total. This represents a significant step towards the feasible manufacturing of large‐scale all‐printed integrated circuits based on OECTs.

Voltage divider‐type unipolar circuits have also seen innovation in the composition of their substrate, leading to unusual circuit properties that could allow for unconventional applications. One example includes a circuit in which the OECT component was a flexible, free‐standing, adhesive composite fabricated from PEDOT:PSS and nanofibrillated cellulose (Figure 4C).^[^ ^60^ ^]^ This composite material combined mechanical robustness (elastic modulus of 4.4 MPa) with good electrical performance (conductivity of 30 S cm^−1^), and its inherent adhesiveness enabled repeated lamination and delamination, allowing for easy assembly and reconfiguration of circuits. The OECTs were connected to stencil‐printed PEDOT:PSS resistors on plastic substrates, where the resistance could be tuned by adjusting the length of the printed strip. The authors demonstrated basic logic gates, including NOT, NOR, and NAND, using this fully organic and reconfigurable approach. While limited in detailed circuit performance metrics, the work highlights a novel direction toward sustainable, modular, and low‐cost manufacturing of electrochemical circuits, with potential for use in transient and flexible bioelectronics.

In another case, PEDOT:PSS based OECTs were built on a flexible gelatin hydrogel substrate, which also served as the electrolyte.^[^ ^67^ ^]^ This dual use of the gelatin hydrogel enabled the fabrication of fully soft and biocompatible circuits, where the electrical characteristics of the OECTs were modulated by the pH of the gelatin electrolyte, allowing for the fabrication of pH‐responsive NOR and NAND gates. The unipolar inverter logic circuit consisted of three resistors (68, 47, 20 kΩ) and a single OECT connected in series, which formed the basis for the NOR and NAND gates and achieved an approximate 1.5 maximum gain. Additionally, the magnitude of the inverter's *V*_out_ could be tuned by varying the pH of the hydrogel electrolyte without altering *V*_in_. Together, the pH‐responsive behaviour and biocompatibility of the components underscore the potential for this kind of circuit to be integrated into bioelectronic devices and advanced pH or ion biosensors. Moreover, using an inverter configuration provides capabilities that a single OECT sensor could not achieve, including multiscale functionality that extends from the detection of small ion concentrations to building integrated sensing platforms. Unlike single OECT sensors, OECT‐based inverters can convert minute ion‐driven variations into full rail‐to‐rail voltage output with defined noise margins. This high‐gain amplification transforms weak biochemical signals, for instance, into strong signal outputs that are less affected by electrical noise and system component drift. This was demonstrated by Torricelli and co‐workers, showing that OECT inverters deliver substantial voltage gain and high noise margins even at sub‐volt supply levels, with these performance metrics being electrolyte‐tuneable through ion buffering and interface charge modulation.^[^ ^44^ ^]^ Furthermore, inverters enable decision‐level outputs (e.g., NAND) and multiplex capabilities with minimal wiring. Logic‐gate sensors can also co‐locate sensing and processing in the same circuit, reducing power and noise pickup compared with analog routing to remote instrumentation.

**Table 1:** Summary of single OECT devices: channel material, electrolyte type, channel geometry, and device performance parameters.

Channel Material	Electrolyte	Thickness [µm]	Width [µm]	Length [µm]	*I*_ON_/*I*_OFF_ ratio	*µ* [cm^2^ V^−1^ s^−1^]	C* [F cm^−3^]	µC* [F cm^−1^ V^−1^ s^−1^]	*g*_m_ [mS]	*V*_Th_ [V]	Switching speed [s] τON and τOFF (ms)	Ref
PEDOT:PSS and nanofibrillated cellulose	Ion‐gel electrolyte	120	—	—	237				45			[60]
PEDOT:PSS	Aqueous liquid electrolyte (range of concentrations)	9	953	417	598–2064	5 × 10^−12^ to 7 × 10^−12^			16.6 – 43.3			[61]
PEDOT:PSS	Ion‐gel electrolyte	–	100	150	≈10^5^	–			–		≈30 (ON) ≈20 (OFF)	[59]
PEDOT:PSS	Aqueous polyelectrolyte solution	0.1	13	6	6.4 × 10^3^	–	–		–	–		[64]
PEDOT:PSS	Ion‐gel electrolyte	0.1	500	1200	–	–	–		0.27	–		[67]
PEDOT:PSS	Aqueous liquid electrolyte	0.4	1000	250	≈10^3^	2.26	37.3	84	12.9	–		[68]
PEDOT:PSS	Aqueous liquid electrolyte	2	1000	500	–	–	44		10	–		[44]
PEDOT‐S:H	Extracellular medium		20–100		≈40	–	–		0.014	–		[70]
PEDOT:PSS	Aqueous polyelectrolyte solution	0.1	1300	160	∼10^3^	–	–		Normalized (S cm^−1^): 70 S	–		[71]
P(g42T‐T) (p); BBL (n)	Aqueous polyelectrolyte solution	0.02(p) 0.25(n)	2000	200	∼10^3^	–	–	22.9 (p) 2.63 (n)	0.17 (p) 0.18 (n)	–	48 (p‐ON) 16 (p‐ON) 145 (n‐ON) 160 (n‐OFF)	[74]
gDPP‐g2T (p); Homo‐gDPP (n) vOECT	Aqueous liquid electrolyte	0.1	vOECT area: 30 × 70		≥10^6^	3.33(p) 3.06 (n)	–		384 (p) 251 (n)	0.10 (p) 0.21 (n)	0.425 (p‐ON) 0.085 (p‐ON) 0.366 (n‐ON) 0.045 (n‐OFF)	[78]
gDPP‐g2T (p); Homo‐gDPP (n) pOECT**			100	10	∽10^3^		–		13 (p) 0.49 (n)			[78]
Cl_2_‐BAL (anti‐ambipolar)	Aqueous liquid electrolyte	0.16	100	5	10^5^	0.014	442	6.2	Normalized (S cm^−1^): 1.63	0.26	2.4 (ON)	[81]
p(C4‐T2‐C0‐EG) (ambipolar)	Aqueous liquid electrolyte	0.05	100	10		1.48×10^−3^ (p) 1.28×10^−3^ (n)	90 (p) – 125 (n)		Normalized (S cm^−1^): 2.73 × 10^−2^ (p) 3.07 × 10^−2^ (n)	−0.6 (p) 0.3 (n)		[87]
gIDT–BBT (ambipolar)	Aqueous liquid electrolyte	–	30	30	10^6^	–	–		170 (p) 34 (n)	−0.15 (p) 0.18 (n)	490 (p‐ON) 120 (p‐OFF) 670 (n‐ON) 570 (n‐OFF)	[88]
gIDT‐BBT (ambipolar) with the crosslinker DtFDA (6:1)									155 (p) 27 (n)	−0.05 (p) 0.16 (n)	170 (p‐ON) 10 (p‐OFF) 36 (n‐ON) 20 (n‐OFF)	[88]
DHF‐gTT (ambipolar)	Aqueous liquid electrolyte	0.12	2000	100	0.4 × 10^3^(p) 0.9 10^3^	0.284 (p) 0.134 (n)	42 (p) 105 (n)	12 (p) 14 (n)	Normalized (S cm^−1^): 2.6 (p) 4.17 (n)	−0.72 (p) 0.28 (n)	38.75 (p‐ON) 5.77 (p‐OFF) 5.87 (n‐ON) 1.57 (n‐OFF)	[89]
p(gDPP‐V) (ambipolar) vOECT	Aqueous liquid electrolyte		vOECT device area:30 × 30		10^7^ (p) 10^7^ (n)	–			Area normalized (µS µm^−^^2^): 297 (p) 294 (n)	−0.18 (p) 0.34 (n)	≺ 2 (p) ≺ 2 (n)	[90]
p(gDPP‐V) (ambipolar) pOECT		0.13	100	10	10^4^(p) 10^5^(n)	1.61 (p) 0.98 (n)	142 (p) 112 (n)	204 (p) 102 (n)	Normalized (S cm^−1^): 29 (p) 25 (n)		63 (p‐ON) 24 (p‐OFF) 41 (n‐ON) 22 (n‐OFF)	[90]
p(g2T‐TT) PrC60MA (ambipolar)	Aqueous liquid electrolyte	0.06	1000	30	>10^3^	–		22.8 (p) 11.8 (n)	Normalized (S cm^−1^): 4.8 (p) 3.0 (n)	−0.09 (p) 0.65 (p)	466 (p‐ON) 5.8 (p‐OFF) 20 (n‐ON) 5.6 (n‐OFF)	[91]
PEDOT:PSS + PEI + sorbitol	Embedded hydrated ions	0.10	5	30		0.1–10	–		5.49	–	0.086	[92]
P(T_0_T_0_TT_16_) (p) P(NDI2OD‐T2) (n)	Aqueous polyelectrolyte solution	0.1–0.04	2.5	1000	10^4^(p) 10^6^(n)	0.03 (p) 0.007 (n)	–		Normalized (S cm^−1^): 18 × 10^−5^ (p) 4 × 10^−5^ (n)	−0.5 (p) 0.1 (n)		[93]
PEDOT:PSS + d‐sorbitol	Embedded hydrated ions	2	12	30	4 × 10^6^	–	–		0.8	–	0.032(ON)	[94]
P(T_0_T_0_TT_16_) (p)	Aqueous polyelectrolyte solution	0.070	3–4	2–3		0.02	–		Normalized (S cm^−1^): 13.5 × 10^−5^	–		[95]
PEDOT:PSS	Ion‐gel electrolyte	–	–	0.05	≈6 × 10^4^	–	–		30	0.8	8 × 10^−5^ (ON) 8.5 × 10^−5^ (OFF)	[58]

For studies that tested a range of operational parameters or device geometries, the best parameters achieved are reported in the table; **pOECT: planar OECT.

### 4.3 Unipolar Circuits: Load OECT Type

An optimal unipolar circuit depends on the characteristics of its components, namely, the OECT and the load element. In this design, using an OECT as the load component allows tunable resistances. Although the load transistor can be fabricated from various materials due to its passive role in the circuit, a useful example to start with is a 3D printed, unipolar inverter logic gate composed of two OECTs, both utilizing PEDOT:PSS as the conductive channel material (Figure 4D).^[^ ^61^ ^]^ To achieve the inverter output characteristics, the load OECT (the transistor closer to *V*_DD_) was gated by the same drive voltage (*V*_DD_ = −0.3 V) as had been applied to its source, maintaining it in its ON state. The gate of the driver transistor, located farther from *V*_DD_, was connected to the input voltage, which tuned the conductivity of the OECT and resulted in the characteristic inverter output. The inverter was successfully implemented as a cation‐type detector and concentration sensor in aqueous electrolytes. A similar unipolar circuit design has been adopted as a voltage amplifier, demonstrating the efficacy of altering the composition of the load OECT by featuring two *p*‐type OECTs, PEDOT:PSS for the load transistor, and p(g2T‐T) as the active material in the drive transistor.^[^ ^68^ ^]^ A maximum gain of ≈18 was achieved at a supply voltage of −0.8 V, indicating efficient amplification under low‐power operation conditions. These results demonstrate that OECT‐based circuits can offer both high signal amplification and reliable logic operation at low supply voltages, making them well‐suited for energy‐efficient bioelectronic and wearable applications.

A unique alternative has been demonstrated to the standard OECT, which showed that the threshold voltage (the minimum gate voltage required for the transistor channel to begin significant conduction, marking the switch from OFF to ON) of a single OECT, can be tuned by replacing the conventional gate electrode with an electrochemical oscillator.^[^ ^69^ ^]^ In this setup, a DC current is applied between the gate and counter‐gate electrodes of the oscillator, inducing electrochemical reactions that modulate the gate's electrochemical potential. This modulation causes a shift in the OECT's threshold voltage, which has been leveraged to implement and optimize simple unipolar inverter circuits. These optimized circuits achieved a voltage gain of up to 20 and a noise margin of 320 mV when operated at a supply voltage of 0.8 V.^[^ ^69^ ^]^ Furthermore, significant advancements have been achieved in the overall performance of unipolar inverter circuits, obtaining a gain larger than 100 by simply tuning the concentration of the electrolyte.^[^ ^44^ ^]^

The interest in OECT‐based logic circuits for bioelectronic applications is underpinned by the vast potential for biological integration that these systems have. As such, significant progress has been made in advancing unipolar OECT circuits toward biohybrid and sustainable applications. One example showed the incorporation of CPs within living plant systems, where a rosa floribunda stem was immersed in a sulfonated PEDOT derivative solution (PEDOT‐S:H), enabling uptake via the xylem and in situ formation of conductive hydrogel wires. These PEDOT‐S:H xylem wires were essentially a conductive CP hydrogel surrounded by the natural electrolyte structures of the rose stem and could therefore function as the active channel material in OECTs that were successfully employed to realize NOT and NOR logic circuits.^[^ ^70^ ^]^ The resulting plant‐integrated circuits operated reliably, with the NOT gate switching between ON at −0.5 V and OFF at −0.3 V, demonstrating a novel form of sustainable bio‐integrated logic. Developments on the opposite end of the device's lifecycle have also been made with fully printed high‐gain OECT inverters and sensors on compostable cellulose substrates.^[^ ^71^ ^]^ The mask‐free, printed, unipolar PEDOT:PSS‐based inverters exhibited a gain of ≈137, and demonstrated real‐time ion detection with a sensitivity up to 506 mV∙dec^−1^. This fabrication approach, based on dispensing and direct‐writing techniques, incorporates environmental safety with monolithic integration of sensing and logic on biodegradable materials, ideal for wearable and disposable applications. Together, these examples underscore the potential of unipolar OECT circuits in realising biointegrated electronic technologies.

**Table 2:** Summary of OECT‐based logic circuits: channel material, type of logic gate, supply voltage (*V*_DD_), gate switching speed, logic gate gain, and power consumption.

Channel material	Logic gate	*V*_DD_ [V]	switching speed [s]	Logic gate gain	Power Consumption [µW]	Refs.
Unipolar Circuit Configuration						
PEDOT:PSS and nanofibrillated cellulose	Inverter; NAND; NOR		30	–	–	[60]
PEDOT:PSS	Inverter	−0.3	–	–	–	[61]
PEDOT:PSS	Inverter; NAND; Decoder; Shift register; Ring oscillator	±5	20 × 10^−3^(NAND) 54 × 10^−3^(Inverter) 150 × 10^−3^(Decoder)	–	–	[59]
PEDOT:PSS	Inverter; Oscillator; NAND; NOR	±3	10	–	–	[62]
PEDOT:PSS	Inverter; Oscillator; NAND; NOR	+1.2	20	–	–	[63]
PEDOT:PSS	Inverter; NAND	+4.3 & −2.1	0.03 (OFF) 0.9 (ON)	3.8	–	[64]
PEDOT:PSS	Inverter; NAND; Flip‐flop; Shift register	±3.5	1.38	3.3	–	[65]
PEDOT:PSS	Inverter; NOR; NAND	±3	–	≈1.5	–	[67]
PEDOT:PSS	Inverter	−0.8	–	18	–	[68]
PEDOT:PSS	Inverter; Oscillator	0.8	–	≈20	–	[69]
PEDOT:PSS	Inverter	0.8	–	107	–	[44]
PEDOT‐S:H	NOR	−1.5	20	–	–	[70]
PEDOT:PSS	Inverter	0.6	–	82	–	[71]
PEDOT:PSS + d‐sorbitol	NAND; NOR	–	2.6 ×10^−6^	–	–	[94]
P(T_0_T_0_TT_16_) (p)	Inverter; Ring Oscillator	1.5	4.2 × 10^−3^ (Oscillator)	–	–	[95]
PEDOT:PSS	Inverter; NAND; NOR	0.5	–	≈3	–	[58]
P3HT	Inverter; NAND; Ring Oscillator; Flip‐flop	−1.5	5 × 10^−5^	7	–	[56]
Complementary Circuit Configuration						
P(g42T‐T) (p) BBL (n)	Inverter	0.7	–	26	2.7	[74]
Polyaniline	Inverter (single‐material)	0.2	–	7.2	–	[75]
gDPP‐g2T (p) Homo‐gDPP (n)	Inverter; NAND; NOR; Ring oscillator	0.7		150	<1	[78]
BBL (ambipolar)	XOR	0.6		–	≈50	[23]
Cl_2_‐BAL (n) p(g1T2‐g5T2) (p)	Reconfigurable AND/NOR/OR/NAND, inverter	0.5	–	35	–	[81]
PEDOT:PSS (p) BBL (n)	AND; NAND; NOR; OR	0.1	–	–	8.2	[83]
p(C4‐T2‐C0‐EG)	Inverter	0.8		28	0.058	[87]
gIDT–BBT (ambipolar)	Inverter	0.9		28	–	[88]
DHF‐gTT (ambipolar)	Inverter	0.8	–	102	–	[89]
p(gDPP‐V) (ambipolar)	Inverter; NAND; NOR	0.8	–	105	–	[90]
p(g2T‐TT) + PrC60MA Blend (ambipolar)	Inverter	0.9	–	82	–	[91]
P(T_0_T_0_TT_16_) (p); P(NDI2OD‐T2) (n)	Inverter; Ring oscillator	1	≈0.3 ×10^−3^	17.5	≺2.5 × 10^−3^	[93]

For studies that tested a range of VDD values, the VDD that resulted in the highest gain is reported in the table.

## 5 Complementary Circuits: Implementation

Complementary circuits, which integrate both *p*‐ and *n*‐type transistors, are fundamental in modern electronics due to their low power consumption, noise suppression, and high signal gain.^[^ ^37^ ^]^ In this section, we review examples of OECT‐based complementary circuits, focusing on whether separate *p*‐ and *n*‐type transistors are used, or whether a single ambipolar or anti‐ambipolar transistor can fulfill both roles.

### 5.1 Complementary Circuits: Discrete *n*‐OECT/*p*‐OECT Type

Following the development of the first OECT by Wrighton and collegues,^[^ ^22^ ^]^ the first investigation into the design of OECT‐based complementary circuits was presented by McCoy et al.,^[^ ^72^ ^]^ who used pairs of microelectrochemical transistors made from different CPs as active materials. These devices functioned as push–pull amplifiers that effectively eliminated crossover distortion, a common issue in traditional amplifiers, thanks to the unique conductivity windows of these CPs. Interestingly, the OECT's transfer characteristics overlapped within a specific voltage range, providing an accessible window for operation. This enabled a complementary configuration where one device was ON when the other was OFF, and vice versa, enabling the operation of a functional complementary circuit. It was not until more than two decades later that CP‐based OECTs were incorporated into complementary circuits.^[^ ^45^ ^]^ Two transistors were connected in series, a high‐performing *n*‐type OECT based on BBL and a *p*‐type transistor using poly‐(3‐carboxy‐pentyl‐thiphene) as the active layer, obtaining the typical inverter characteristics with a gain of up to 12. A subsequent study reported a complementary NOT logic circuit that acted as an amplifier, in which the *p*‐type OECT was composed of PEDOT:PSS and the *n*‐type OECT was made of BBL. Exploiting the ion‐to‐electron transduction capability of OECTs, the amplifier demonstrated selective ion detection while also providing high signal amplification.^[^ ^73^ ^]^ Increasing the ion concentration in the electrolyte caused a shift in the transfer characteristics of the NOT circuit toward lower potentials, with a sensitivity exceeding 2300 mV V^−1^ dec^−1^, orders of magnitude higher than that of previously developed sensing devices. The biomedical application of the circuit was demonstrated by real‐time detection of potassium in human serum.

**Figure 5:**
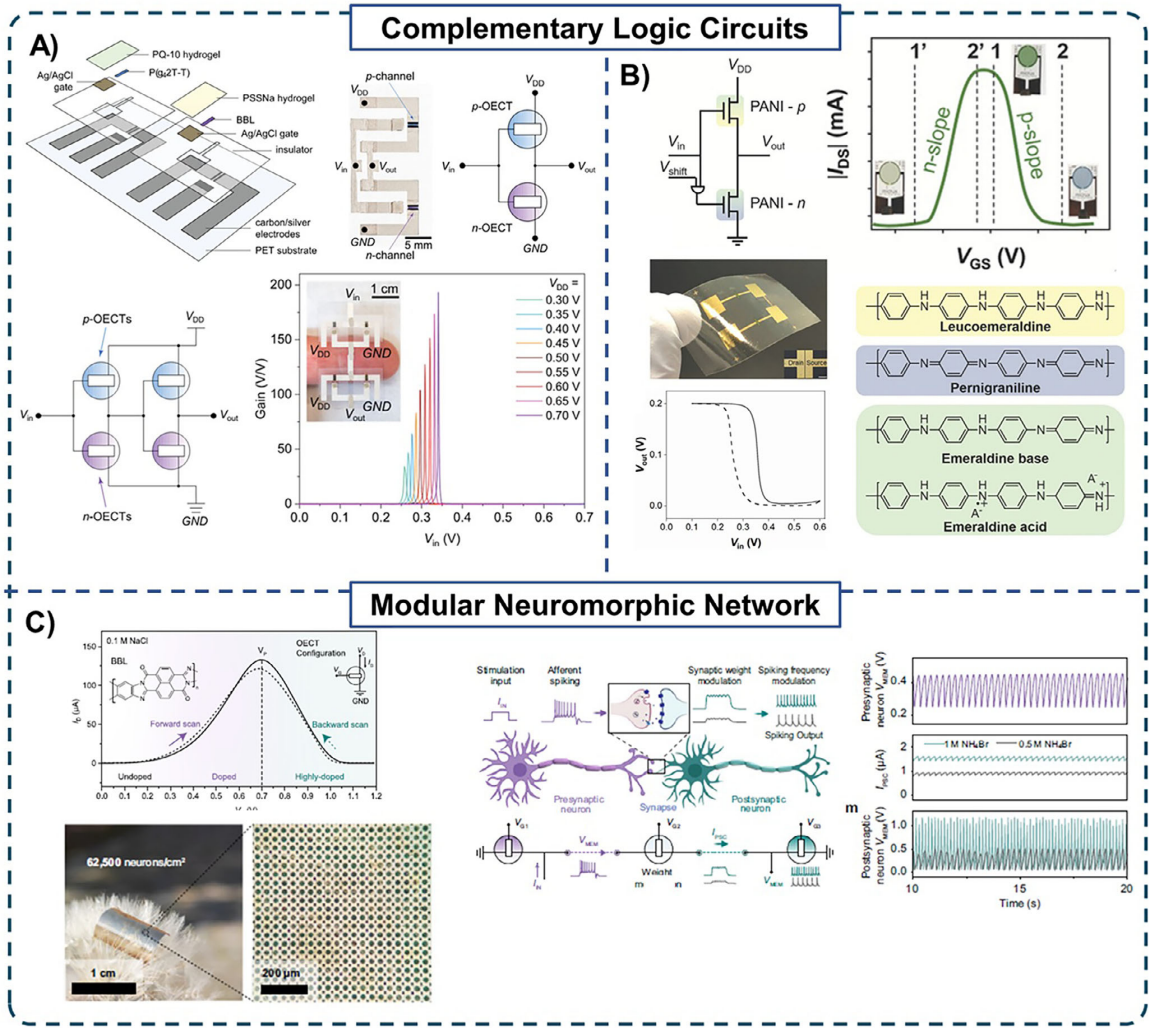
A) Schematic illustration of layer‐by‐layer fabrication of complementary OECTs. Photograph and schematic layout of a printed complementary inverter. Schematic of a monolithically integrated two‐stage amplifier and its voltage gain characteristics. *Inset*: Photograph of the fully printed two‐stage complementary OECT amplifier. Reproduced with permission.^[^ ^74^ ^]^ Copyright 2021, Wiley. B) Schematic of the first demonstration of a flexible complementary inverter using a single active material, polyaniline (PANI), in both channels. Transfer curve showcasing the unique bell‐shaped transfer characteristics that arise from the distinct redox states of PANI (chemical structures shown), enabling the same material to function as both a *p*‐ and *n*‐type transistor. A digital image showing the circuit fabricated on a flexible chitosan substrate and the voltage transfer characteristic of the inverter. Reproduced with permission.^[^ ^75^ ^]^ Copyright 2020, Wiley. C) Anti‐ambipolar transfer characteristic of a BBL‐based OECT and a photograph of BBL‐OECT array integrating 625 000 OECTs cm^−2^. A modular neuromorphic system consisting of three OECTs, with spiking from the pre‐synapse neuron, modulated by the synapse neuron, and transmitted to the post‐synapse neuron, where it is converted to an output signal. Continuous voltage spiking waveform (*V*_MEM_) shown as a function of each synapse. Reproduced with permission.^[^ ^76^ ^]^ Copyright 2025, Springer Nature.

The fabrication feasibility of flexible and low‐power complementary circuits has been demonstrated using printing techniques.^[^ ^74^ ^]^ The circuit consisted of *p*‐ and *n*‐type enhancement‐mode OECTs based on p(g_4_2T‐T) and BBL, respectively (**Figure** **5**A). Through a planar side‐gate design and hydrogel electrolytes tailored for each polarity, high power‐efficient complementary voltage amplifiers operating below 1 V were achieved. Their single‐stage amplifier was capable of sensing signals as low as 100 µV with a gain of 30.4 dB, while the two‐stage configuration reached a record DC gain of 193.

Innovative manufacturing methods can also play a major role in the future of OECT‐based logic. For example, a femtosecond laser micropatterning method has been demonstrated for PEDOT:PSS and other CPs, achieving >100× faster switching due to the sub‐5 µm feature size.^[^ ^77^ ^]^ This cleanroom‐free technique enabled rapid and high‐resolution fabrication of OECT complementary inverters and glucose biosensors, and was compatible with both *p*‐ and *n*‐type devices. As noted by the authors, such a rapid and versatile fabrication technology could significantly accelerate the exploration of OECT‐based logic.

While the previous examples used planar OECTs, complementary circuits have also been demonstrated using vOECTs, in which the *n*‐type OECT was directly deposited on top of the *p*‐type OECT.^[^ ^78^ ^]^ This 3D device architecture was fabricated using conventional techniques; the vertical configuration is appealing as it yields higher integration density, allowing a larger number of transistors to be connected within a compact area. The complementary circuit exhibited a significant voltage gain (≈150) with a sharp voltage transition, enabled by the fast‐switching times of the individual OECT devices, 425 µs for the *p*‐type and 366 µs for the *n*‐type, showing that the two polymers had balanced transport properties.

The dearth of examples of complementary logic circuits, as well as more complex circuits based on *n*‐ and *p*‐type CPs, is a result of mismatched material availability. CPs are mostly *p*‐type, and while OECTs with this active layer exhibit high performance, the exceptionally limited selection of *n*‐type CPs with comparable performance limits the fabrication of circuits that require well‐matched transport properties.

### 5.2 Complementary Circuits: Ambipolar and Anti‐Ambipolar

Approaches that involve changes in OECT channel dimensions and active layer thickness can be used to obtain matching performance OECTs; however, this drastically increases the fabrication process complexity and device footprint. An alternative design to mitigate these issues while retaining the benefits of the complementary circuit is the ambipolar and anti‐ambipolar complementary circuits, wherein carefully designed OECTs with unique operational windows allow for a circuit with one material type to function in a complementary mode.

The first complementary circuit using only one material as an active channel in both OECTs was reported in 2021.^[^ ^75^ ^]^ Inspired by the first push and pull amplifier presented by Paul et al.,^[^ ^79^ ^]^ PANI was selected as the active layer for its exceptional electrochemical characteristics, specifically the peak in its transfer curve (drain current vs *V*_G_) when used as the active OECT channel operating in aqueous electrolytes. Unlike most other CPs, which generally either have an increase or decrease in current with increasing *V*_G_ when operated in an aqueous electrolyte, PANI's three oxidation states result in a rise in current followed by a decrease, resulting in a bell‐shaped transfer curve (OFF–ON–OFF) with an electrochemical window accessible in water. This enabled the design of a complementary circuit using identical OECTs (Figure 5B). The input voltage was applied to the gate of the OECT closer to *V*_DD,_ and the input of the second OECT was shifted by a DC offset equal to −0.4 V. In this configuration, when one OECT was in its ON state, the other was in the OFF state, enabling complementary circuit operation. A maximum gain of ≈7 was achieved when using an inverter configuration for low drive voltages of −0.2 V. The technology was then successfully transferred onto a flexible biocompatible chitosan substrate, demonstrating optimal circuit functionalities in aqueous electrolytes. The bell‐shaped transfer curve shown in PANI was also demonstrated for other polymers operating in aqueous electrolytes, such as BBL^[^ ^23^, ^76^, ^80^ ^]^ and poly(benzimidazoanthradiisoquinolinedione) (BAL),^[^ ^81^ ^]^ where it was described as anti‐ambipolar.^[^ ^80^ ^]^ This behaviour has been explained by charge–charge interactions that occur at high doping levels of CPs.^[^ ^72^, ^82^, ^83^, ^84^ ^]^ Initially, as the gate voltage is applied, charge carriers (holes or electrons) are formed in the polymer semiconductor, resulting in increased conductivity and the characteristic rise in current in the transfer curve (OFF → ON). With further increase in *V*_G_, the doping level increases, and polarons can pair to form bipolarons (multiply charged species), filling localized electronic states within the polymer.^[^ ^83^ ^]^ The accumulation of these multiply charged species leads to strong Coulombic repulsion, limiting carrier mobility. Consequently, despite the high doping level, the channel conductivity reduces, resulting in the current drop that defines the second OFF state (ON → OFF).

The anti‐ambipolar behaviour could be manipulated by electric and chemical means, such as varying *V*_DS_, type of gate electrode, and type and concentration of electrolyte. For instance, the ion modulation of the BBL anti‐ambipolar behaviour has been exploited for developing logic circuits. In one example, PEDOT:PSS (*p*‐type) was layered on top of BBL (*n*‐type) in a vOECT geometry and was shown to exhibit anti‐ambipolar transfer characteristics. The anti‐ambipolar vOECT was then used as the building block to develop anti‐ambipolar OR, NAND, AND, and NOR gates, as well as a Hodgkin–Huxley (HH) neuron model which mimicked the behaviour of a retinal neuron.^[^ ^83^ ^]^ Recent studies have increasingly supported the role of BBL‐based OECTs in developing artificial neurons.^[^ ^23^, ^76^, ^80^, ^85^ ^]^ Artificial neurons are electronic devices that emulate the behaviour of biological neurons and are the building blocks of neuromorphic devices designed to replicate neural network processing capabilities.^[^ ^86^ ^]^ Conventional artificial neurons based on inorganic semiconductors rely on purely electronic mechanisms, which pose challenges in their efficient integration at the biointerface that relies on ionic signaling. Anti‐ambipolar OECTs offer a transformative solution: they operate at low voltages through ion‐driven doping/dedoping of their channel material and could communicate with spike‐like signals, which matches how neurons encode information.^[^ ^23^, ^75^ ^]^ Leveraging the anti‐ambipolarity behaviour observed in the BBL‐based OECT, event‐based sensing and real‐time processing have been demonstrated.^[^ ^76^ ^]^ Unlike unipolar OECTs that exhibit monotonic increase or decrease in peak current, the inherent anti‐ambipolar behaviour eliminates the requirement for additional circuit components such as resistors and capacitors, thereby simplifying circuit architecture and scalability. This was demonstrated by constructing a modular neuromorphic network consisting of only 3 OECTs that achieved stimulus sensing, processing, and spike generation (Figure 5C). Further, scalability was validated by fabricating a high‐density array of the BBL‐based vOECTs (625 000 OECTs cm^−2^) on a flexible parylene substrate, yielding a device footprint of 177 µm^2^. The simplified circuit architecture using anti‐ambipolar OECTs has also been shown using the ladder‐type conjugated polymer, BAL. Encouraged by its exceptional long‐term stability in aqueous environments, maintaining performance over 50 000 operational cycles, and a fast transient response (≈0.56 ms µm^−^^2^), the anti‐ambipolar behaviour of BAL‐based OECTs was exploited for the construction of reconfigurable complementary circuits, capable of dynamically switching between multiple logic gate functions (AND, OR, NOR, and NAND) by simply adjusting the input voltages. This approach significantly streamlines circuit design and pushes the boundaries of dynamic and multifunctional organic electronics.^[^ ^81^ ^]^

The origin of the anti‐ambipolar term stems from ambipolar, which is seen commonly in inorganic electronics. Organic ambipolar OECTs have also been used to realize complementary circuits. The OECT can be either comprised of electron‐donor acceptor type polymers or a blend of electron acceptor and donor materials. One of the first examples of ambipolar complementary circuits was based on a vOECT featuring a polymer with a backbone consisting of naphthalenetetracarboxylic diimide and bithiophene units as the active channel material.^[^ ^87^ ^]^ A compact ambipolar complementary inverter was developed using two of these vOECTs connected in series, with a demonstrated gain of 28. This ambipolar inverter was employed as an analog preamplifier for electrocardiogram signal acquisition, demonstrating a tenfold enhancement in signal amplitude compared to direct voltage recordings. Using a similar vertical design approach, an ambipolar inverter circuit with similar gain was developed using the indacenodithiophene (IDT)‐based ambipolar polymer, gIDT–BBT, as the active material instead.^[^ ^88^ ^]^ Copolymerizing a functional bithiophene monomer with a fluorinated bisistain‐lactone, a strong electron‐withdrawing unit, yielded an ambipolar polymer, DHF‐gTT, with high performance when used as an active channel material in OECTs.^[^ ^89^ ^]^ The resulting OECTs demonstrated high and symmetric hole and electron mobilities (≈1.0 cm^2^ V^−1^ s^−1^), and high stability in ambient conditions. Implementing these OECTs as the building blocks in ambipolar inverter configurations, a voltage gain exceeding 100 was achieved. The polymer p(gDPP‐V), consisting of the electron‐acceptor diketopyrrolopyrrole and the electron‐donating vinylene units, is another example of an ambipolar polymer used in circuit design.^[^ ^90^ ^]^ Flexible inverter circuits fabricated by printing were achieved with a gain >100, and successfully employed for real‐time electrooculogram signal monitoring during eye movements. The low‐amplitude input signal from the eye (1.5 mV) was amplified by the inverter, yielding an output signal of 95 mV, validating the capability of these circuits for bio‐signal acquisition and processing. Additionally, the ambipolar OECT was used to construct basic logic gates (NAND, NOR), exhibiting reliable switching responses across multiple *V*_in_ combinations. While the previous examples were based on electron‐donor acceptor polymers, ambipolar OECTs and inverters have also been realized using a bulk‐heterojunction blend of a *p*‐type polythiophene (p(g2T‐TT)) and an *n*‐type fullerene derivative (PrC_60_MA).^[^ ^91^ ^]^ Using a blend of an electron acceptor and an electron donor can offer flexibility in independently tuning the properties of individual components and can also be considered a simpler approach than the synthesis of donor‐acceptor polymers.

## 6 OECT Circuit Design Beyond Conventional Liquid Electrolytes

**Figure 6:**
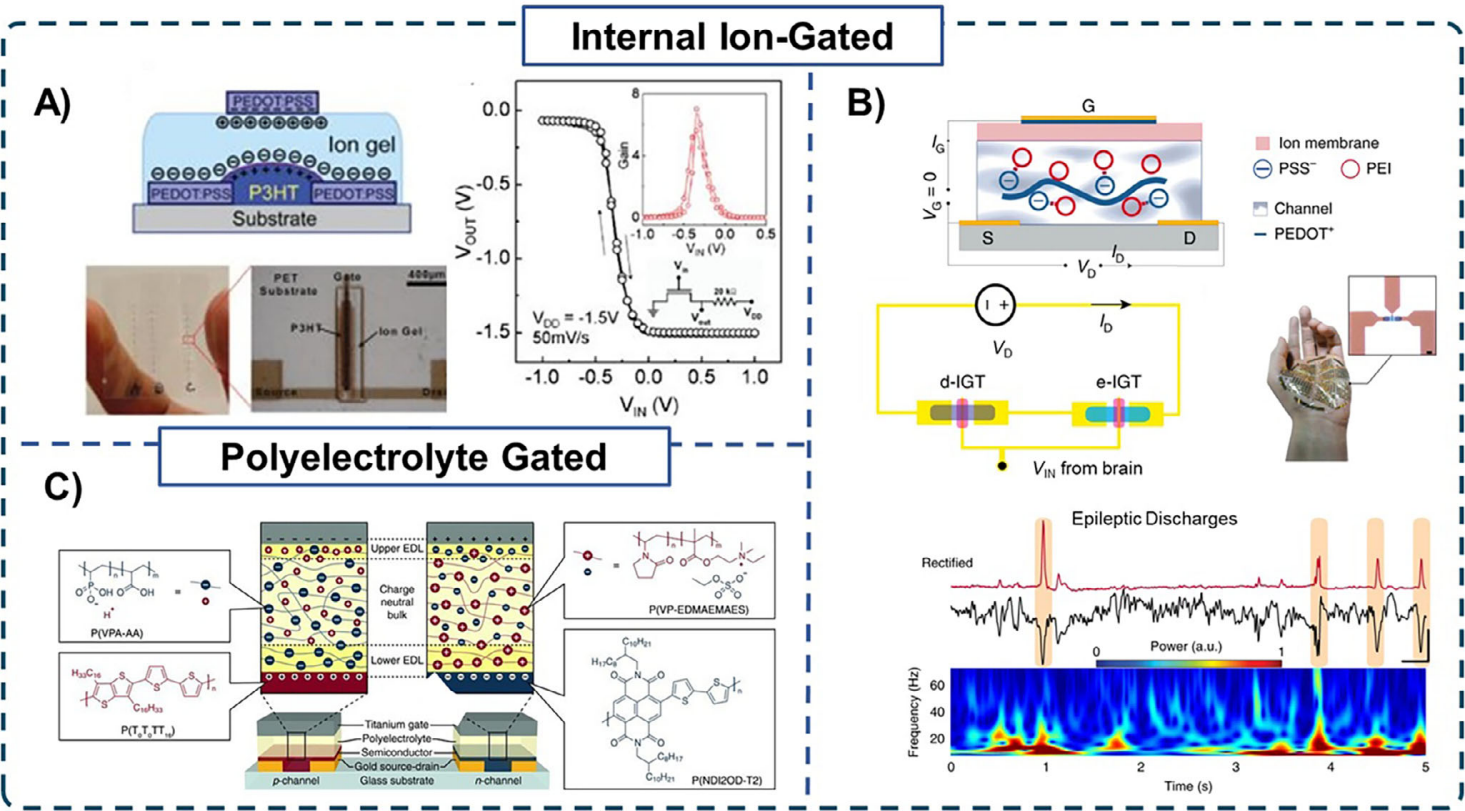
A) Diagram and image of an all‐printed ion‐gated P3HT transistor having PEDOT:PSS as a gate, and its circuit voltage characteristics when connected in series with a load resistor. Reproduced with permission.^[^ ^56^ ^]^ Copyright 2010, Wiley. B) Schematic of an IGT cross‐section in which D‐sorbitol forms a hydrated ion reservoir in the channel. Digital image of the flexible, thin e‐IGT array conforming to a human hand. Schematic of a non‐linear rectification circuit made from IGT, which enabled high signal‐to‐noise real‐time detection of epileptic discharges. Reproduced with permission.^[^ ^92^ ^]^ Copyright 2020, Springer Nature. C) Diagram of materials system for complementary polyelectrolyte‐gated transistors. Schematic cross‐section shows the charge distribution within the chemical composition of the polyelectrolyte gate insulator layers in the *p*‐ and *n*‐channel transistors gated by negative and positive gate voltages, respectively. Reproduced with permission.^[^ ^93^ ^]^ Copyright 2024, Wiley.

While logic circuit layouts may vary, the performance of such circuits is strongly governed by the operational characteristics of their OECT components. Exploring alternative design strategies expands the possibilities for constructing advanced logic circuits with enhanced functionality and performance. A highly effective OECT design strategy involves using ion‐gel or embedding mobile ions within the CP matrix of the OECT's active layer to create an internal ion‐gated transistor (IGT), rather than relying on a liquid electrolyte in direct contact with the semiconductor. This approach offers several advantages, including increased ion mobility, faster switching times, and improved transconductance. By shortening the distance the ions need to travel before interacting with the CP, this design strategy enhances the overall performance of the OECT and ultimately the logic circuit. Furthermore, it provides a promising pathway to scalability compared to liquid electrolytes, enabling more compact device geometries for large‐scale circuit integration. One of the first examples of an IGT was a poly(3‐hexylthiophene) transistor with an ion‐gel top layer composed of a mixture of ionic liquid (1‐ethyl‐3‐methylimidazolium bis(trifluoromethylsulfonyl)imide) and a gelating triblock copolymer (poly(styrene‐*b*‐methylmethacrylate‐*b*‐styrene)) (**Figure** **6**A). The ion‐gel‐gated transistor was fully fabricated using aerosol‐jet printing, a low‐cost technique that resulted in reproducible and uniform devices. The ion‐gel‐gated transistor exhibited a low threshold voltage (≈0 V) with the current increasing 5 orders of magnitude at *V*_G_ = −1 V. The resulting transistors were integrated into unipolar NOT and NAND gates, ring oscillators, and flip‐flop circuits, achieving reliable circuit performance.^[^ ^56^ ^]^ Further, the study demonstrated the fabrication of an all‐organic inverter using a 20 kΩ printed PEDOT:PSS resistor connected in series with the ion‐gel gated transistor, resulting in a gain of 7 and showing no hysteresis upon sweeping the input voltage.

More recently, a vertical architecture has been leveraged to fabricate an ion‐gel‐gated vOECT consisting of PEDOT:PSS as the channel material and 1‐ethyl‐3‐methylimidazolium bis(trifluoromethylsulfonyl)imide/poly(ethylene glycol) diacrylate as the ion‐gel deposited on the surface. The vOECT could be switched on in 80 ns, exhibiting exceptional performance with a record operational frequency of 12 MHz V^−1^. This was not only enabled by the residence of the ionic species inside the channel but also by the vertical stacking of the solid Ag/AgCl gate that was spray‐coated on top of the ion gel. This design strategy is compatible with scalable integrations, which was demonstrated by fabricating various logic gates with reliable operations.^[^ ^58^ ^]^

Following a similar principle, mobile ions could be incorporated within the bulk of the channel material. For example, incorporating D‐sorbitol, a hydrophilic sugar molecule, in a PEDOT:PSS active channel material aided the uptake of water, which in turn facilitated ion movement within the channel, and created an ion reservoir. To avoid parasitic current from the gate, a layer of chitosan that acted as an ion membrane was deposited between the gate electrode and the active layer of the transistor.^[^ ^94^ ^]^ Due to the shortening of the transit time of the mobile ions, the IGT showed higher transconductance and faster rise time (31.7 µs) compared to an electrolyte‐gated device (191.2 µs). The IGT was then successfully used to capture high‐quality neurophysiological data from a human participant. Additionally, the IGT design could be readily incorporated into flexible, conformable ICs, including unipolar NAND and NOR digital gates and cascade amplifiers. Another IGT, this time in enhancement mode (meaning it was in the OFF state at *V*_G_ = 0 V), used D‐sorbitol and PEDOT:PSS with the addition of polyethylenimine as the composite for the active layer (Figure 6B).^[^ ^92^ ^]^ The device exhibited a high transconductance and speed (2.9 µs rise time), which was several orders of magnitude faster than previously reported enhancement‐mode transistors. These exceptional performance characteristics facilitated the acquisition of a diverse range of electrophysiological measurements, including electromyography, electrocardiography, and encephalography in both humans and rats. The IGT operated effectively over a range of physiological frequencies (0.1–10^4^ Hz) while maintaining low noise and high signal‐to‐noise ratios. The IGT was also incorporated into unipolar AND and OR gate configurations, successfully implementing the corresponding logic functions.

Polyelectrolyte‐gated OECTs provide an alternative to IGTs. Unlike IGTs, which incorporate mobile ions directly within the CP matrix and operate without the need for ion exchange with an external electrolyte, polyelectrolyte‐gated devices use a separate polyelectrolyte layer as the gate dielectric. When a voltage is applied to the gate electrode, the mobile ions in the polyelectrolyte redistribute and form electric double layers at both the gate–electrolyte and electrolyte–semiconductor interfaces, with a charge‐neutral region in between. The resulting capacitive coupling enables efficient modulation of charge carrier density in the semiconductor without requiring ion penetration into the active layer. As a result, these devices offer improved stability and charge control, making them well‐suited for integrated logic circuits. This strategy was adopted to fabricate complementary inverters using a polyanion to gate a *p*‐type CP and a polycation to gate an *n*‐type OECT (Figure 6C).^[^ ^93^ ^]^ The transistors operated in accumulation mode (OFF at *V*_G_ = 0 V) so that the mobile ions in the polyelectrolyte were attracted to the gate electrode and depleted from the CP/polyelectrolyte interface. Thus, an upper electric double layer was formed at the interface with the gate electrode, and a lower electric double layer was formed at the CP/polyelectrolyte interface. As the charges could not penetrate into the bulk of the CP channel, there was no electrochemical doping, and the transistors operated in a field‐effect mode. These material combinations formed the basis for the corresponding OECTs demonstrated in a complementary inverter and ring oscillator, which exhibited operating voltages as low as 0.2 V and a static power dissipation of less than 2.5 nW for the logic gate. In a similar polyelectrolyte‐gated approach, Herlogsson et al. demonstrated integrated circuits based on organic transistors with active channels made of a polythiophene derivative gated by the anionic polyelectrolyte, poly(vinyl phosphonic acid‐co‐acrylic acid).^[^ ^95^ ^]^ These enhancement‐mode transistors were employed in two inverter circuits: a saturated load transistor, where the gate is connected with its drain, and a depleted load, where the gate is connected with its source. Subsequently, a seven‐stage ring oscillator was fabricated using the saturated load inverter design, obtaining a circuit oscillation with a frequency of 235 Hz with 1 V drive voltage.

## 7 Conclusions and Outlook

Leveraging the unique mixed ionic‐electronic conduction mechanism of OECTs offers a promising foundation for developing logic circuits compatible with bioelectronic environments. Their performance can be finely tuned through device geometry and active material selection to meet the demands of low‐voltage operation, ionic signal transduction, and signal amplification. We have presented here a comprehensive overview of design strategies for unipolar and complementary circuits, as well as the implementation of logic circuits using OECTs as the building blocks. By combining multiple OECTs in series or parallel, logic circuits have been successfully developed, enabling the implementation of basic logic gates and more complex circuitry. In unipolar implementations, OECTs benefit from simplified circuit design requiring only one transistor type and easier material optimization, being *p*‐ or *n*‐type. Additionally, the circuit's output voltage can be tuned by the choice of the load component. OECT‐based unipolar circuits have been successfully utilized for their logic function and voltage amplification. Complementary architectures showcased their ability to provide reduced manufacturing complexity, high‐fidelity signal processing, and improved noise margins. These advantages are similar to CMOS technology but with the added benefits of biocompatibility and mechanical flexibility. Of interest here is the recent research into anti‐ambipolar CPs, unravelling their unique advantages for logic gates, primarily through their characteristic transfer curves that exhibit OFF–ON–OFF within a single device. This enables a smaller circuit footprint and simplified fabrication processes, since a single anti‐ambipolar OECT can replace multiple conventional transistors required to achieve similar nonlinear responses. Additionally, the anti‐ambipolar characteristic of these polymers enables the development of electrically reconfigurable logic gates, achieved simply by modulating the input voltage to access both switching characteristic sides of the bell‐shaped curve. This feature represents a step forward toward implementing complex logic operations with minimal components, thereby enhancing processing capability while reducing both physical footprint and energy consumption.

Despite these promising attributes, several challenges remain that can impede the technological advancement of OECT‐based logic circuits, and thus, future research and development in this field should focus on several areas. The switching speeds demonstrated present challenges for high‐speed digital processing applications and real‐time signal conditioning that might require faster response times. The ionic processes in OECTs typically have intrinsic time constants in the millisecond regime, restricting operation frequencies to the kHz range. While this frequency range aligns well with most biological signals, such as neural activity, cardiac rhythms, and muscular contractions, it becomes a limitation in closed‐loop therapies where the processing logic might need to operate at much higher frequencies to analyze complex signal patterns in real‐time and trigger immediate responses. Careful engineering of the device, for example, by tuning the channel material's thickness or optimizing the applied bias, has demonstrated improvement in switching speed down to the tens‐of‐microseconds. Vertical architecture is also a promising design approach to improve current density, transconductance, and switching times. Nevertheless, low‐frequency operation remains broadly the optimal regime.

Organic semiconducting materials, with their complex molecular structures and sensitive processing requirements, must be carefully controlled to achieve consistent electrical performance. This complexity can also result in device‐to‐device variability, hampering their manufacturing at scale. Refining fabrication techniques, such as printing technologies and nanoimprint lithography, can contribute to the fabrication and scalability of these circuits. Long‐term operational stability remains a concern, as many CPs are susceptible to over‐oxidation, potentially leading to threshold voltage shifts and reduced operational lifetime. Additionally, the development of balanced complementary circuits is hindered by the performance asymmetry between *p*‐ and *n*‐type OECTs, where achieving matched threshold voltages, switching speeds, and current drives requires careful material engineering and often results in compromised overall performance. The areas of ambipolar and anti‐ambipolar OECT‐based logic circuits are a quite recent development, and the findings to date are grounds for further investigation. However, while ambipolar OECTs that can operate as both *p*‐ and *n*‐type devices offer potential solutions for simplified complementary circuit designs, they often suffer from mismatched electron and hole mobilities and higher static power consumption. Anti‐ambipolar OECTs present unique opportunities for novel logic architectures but remain largely unexplored for practical circuit applications.

The volumetric nature of OECT operation, where the entire channel contributes to switching rather than just a surface interface, provides exceptional amplification characteristics and allows for effective signal processing in aqueous environments. Therefore, the strategic importance of advancing OECT logic technology extends far beyond incremental improvements in conventional electronics; these devices could be the driving force to progress fully flexible organic bioelectronics into the clinic. As we move toward ubiquitous computing, biointegrated electronics, and sustainable technology solutions, OECTs offer capabilities that silicon‐based systems fundamentally cannot provide. Their mixed ionic‐electronic conduction opens possibilities for in vivo monitoring systems, implantable therapeutic devices, and real‐time biological signal processing applications. The mechanical flexibility and biocompatibility of OECTs make them ideal candidates for wearable health monitors, soft robotics, and human–machine interfaces that require seamless integration with biological tissues. Furthermore, the low‐temperature, solution‐based processing of conjugated polymers offers a sustainable approach and could enable large‐scale manufacturing.

The development of efficient OECT logic circuits is, therefore, a critical technological milestone that could unlock several applications such as neuromorphic computing, advanced biosensing, and bio‐hybrid circuits. In neuromorphic systems, individual OECTs serve as synaptic elements that exhibit short and long‐term plasticity, attributed to time‐dependent ion migration and redistribution processes that mirror biological neural processing. OECT‐based synapses have been shown to generate spike‐like signals at sub‐1 V in physiological media and implement neuronal functions with tunable firing rates and adaptive learning capabilities. Configuring multiple OECTs in logic circuits would create neuromorphic architectures capable of real‐time learning, temporal pattern recognition, and energy‐efficient computation similar to the performance characteristics of biological neural networks. Beyond exploiting their ionic operational mechanism for computation and learning, the sensitivity of OECTs to external ions and biochemical changes can be exploited in biosensing applications. OECT logic circuits enable on‐chip signal processing, amplification, and multiplexing, enhancing sensor performance while reducing the complexity of the circuit. Such biosensors facilitate real‐time signal detection, noise filtering, and multi‐analyte detection directly at the sensing site, eliminating the need for external processing units. This reduced circuit complexity is particularly critical for wearables and bioelectronic implants, which require energy efficiency and rapid response times. The ease of integrating OECTs on flexible substrates is a further appeal for their use in continuous health monitoring devices. In a recent trend, hybrid OECT circuits are leveraging biological entities such as electroactive bacteria to modulate the transistor channel via extracellular electron transfer. Gene expression of electroactive bacteria can be engineered to perform logic operations (NAND, NOR, etc.), translating directly into OECT signals and turning living computation into electrical outputs. The transcriptionally controlled extracellular electron transfer confers synaptic‐like plasticity to the device, pointing to biohybrid sensing, logic, and neuromorphic interfaces. These living logic systems have unique advantages, including self‐regenerating functionality, adaptive reconfigurability, and sensing and computing in a complex biological environment, positioning them as promising platforms for next‐generation circuits.

While their potential in performing reliable logic operations has been demonstrated across several applications, translating OECT‐based logic circuits from benchtop to implantable or wearable devices faces complex constraints at the device/electrolyte/tissue interface. A key step toward the commercialization of implantable devices will be in vivo testing, which is critical given that long‐term exposure to physiological fluid might result in fouling of the CP channel material and introduce drift and hysteresis. The intrinsic operational stability of the conjugated polymer should be evaluated, as continuous electrochemical switching and exposure to reactive biological media can lead to material degradation, reducing device lifetime and performance reliability. Additionally, in vivo testing is critical to collect long‐term safety data to assess biocompatibility, immune response, and toxic effects, all critical for regulatory approval of implantable devices. For wearable applications, devices should be evaluated for long‐term wearability by human participants to demonstrate performance resilience to mechanical stress, sweat, and ambient environmental conditions. Another critical bottleneck for their commercialization is the scalability of fabrication and achieving the high integration density required for complex logic circuits in confined spaces. While advances have been made in fabricating screen‐printed platforms that integrate hundreds of OECTs to realize inverters, 4‐to‐7 stage decoders, and shift registers, their functionality has only been demonstrated in driving simple display/indicator functions, falling short of the complex computing capabilities needed for multifunctional bioelectronic systems. Achieving higher functionality requires far denser integration and a smaller device footprint, which could be attained, for example, by vertical stacking using photolithographic microfabrication techniques. Recent additive manufacturing routes, such as maskless micro‐dispensing and direct printing, offer a pathway toward scaling up the production of integrated circuits. While these advances address technical challenges, economic barriers related to production scale must also be overcome to enable successful commercialization. High‐performance CPs used in OECTs are expensive and currently produced only in small quantities, creating a material cost barrier and limiting market accessibility. Additionally, OECT device fabrication has been confined to research laboratories and small‐scale facilities. Commercialization will require access to high‐volume manufacturing infrastructure that can be very costly, unless existing fabrication processes and production lines currently used in the flexible electronics industry are leveraged. As insurmountable as these challenges appear, nevertheless, the inorganic semiconductor industry overcame similar developmental challenges in its infancy in order to become ubiquitous. Just as today's ultra‐integrated inorganic electronics grew from the very first rudimentary transistor, made of two gold point contacts crudely pressed onto a block of a germanium crystal, OECTs can follow a similar path from simple building blocks to complex, reliable circuits.

## Conflict of Interest

The authors declare no conflict of interest.
